# Mechanism and risk factors of nausea and vomiting after TACE: a retrospective analysis

**DOI:** 10.1186/s12885-021-08253-1

**Published:** 2021-05-07

**Authors:** Haohao Lu, Chuansheng Zheng, Bin Liang, Bin Xiong

**Affiliations:** Department of Radiology, Wuhan Union Hospital, Tongji Medical College, Huazhong University of Science and Technology, Jiefang Avenue #1277, Wuhan, 430022 China

**Keywords:** Nausea and vomiting, TACE, Risk factors, Regression analysis, Hepatocellular carcinoma, Embolic syndrome, Retrospective analysis

## Abstract

**Purpose:**

The mechanism of postoperative nausea and vomiting after TACE is not clear. This study retrospectively analyzed the patient data to explore the mechanism and risk factors of postoperative nausea and vomiting after TACE.

**Materials and methods:**

The data of 221 patients who underwent TACE in the interventional department from January 2019 to December 2020 were collected. Including: gender, age, liver function before TACE, etiology of liver cirrhosis, BCLC stage of hepatocellular carcinoma, preoperative use of analgesic drugs, preoperative limosis, previous history of vomiting, history of kinetosis, smoking history, history of drinking, chemotherapeutic drugs used during TACE, Dosage of lipiodol, and occurrence of postoperative nausea and vomiting.

**Results:**

There were 116 cases of nausea after TACE, using binary logistic regression analysis, Sig: ALT0.003; ALP0.000; history of vomiting 0.043; kinetosis 0.006; history of alcohol consumption 0.011; preoperative limosis 0.006; dosage of lipiodol (5–10 mL) 0.029, dosage of lipiodol (> 10 mL) 0.001.There were 89 cases of vomiting after TACE, all accompanied by nausea, Sig: ALP0.000; BCLC stage (B) 0.007; kinetosis 0.034; chemotherapeutic drugs 0.015; dosage of lipiodol (5–10 ml) 0.015, dosage of lipiodol (> 10 ml) 0.000; patients used analgesics before TACE 0.034.

**Conclusions:**

Causes of post-TACE nausea and vomiting included operative trauma, aseptic inflammation caused by ischemia and hypoxia, chemotherapeutic drugs, ischemia of liver and bile duct, stress and pain during TACE, and patient factors. ALP, BCLC stage, kinetosis, chemotherapeutic drugs, dosage of lipiodol, and preoperative usage of analgesics were risk factors affecting nausea and vomiting after TACE.

## Introduction

Primary hepatocellular carcinoma is one of the malignant tumors with high morbidity and mortality worldwide [1–3], and because there are no obvious specific symptoms and signs in the early stage of the disease, most patients have lost the chance of surgery when detected. Transarterial chemoembolization (TACE) is currently one of the effective treatments for advanced hepatocellular carcinoma [4, 5]. TACE was shown to improve median survival from 16 to 20 months [6]. Its main principle is to superselectively intubate the catheter into the feeding artery of the tumor after establishing a vascular access through femoral artery puncture and inject chemotherapeutic drugs and embolic agents [7]. On the one hand, chemotherapeutic drugs induce apoptosis and inhibit tumor cell proliferation; on the other hand, after tumor supply artery embolization, it leads to tumor cell ischemia, hypoxia and necrosis. TACE is effective in the treatment of liver cancer and plays a very important role in the treatment of hepatocellular carcinoma [8]. The most common side effects after TACE are embolic syndrome, including pain, fever, nausea and vomiting [9–11]. Malignant vomiting, in particular, is one of the major problems faced in clinical practice, which needs to be paid attention to by patients and doctors. Nausea and vomiting will increase the psychological and physical burden of patients, increase the suffering of patients and reduce the compliance of patients with treatment. Severe nausea and vomiting, can lead to water and electrolyte imbalance in patients, prolong hospital stay, and increase treatment costs [12]. At the same time, patients with hepatocellular carcinoma are mostly associated with cirrhosis and gastric esophageal varices, and severe nausea and vomiting will lead to gastric esophageal variceal bleeding, leading to death of patients. The mechanism of postoperative nausea and vomiting after TACE is not clear. This study retrospectively analyzed the patient data to explore the mechanism and risk factors of postoperative nausea and vomiting after TACE.

## Materials and methods

### General information

The data of 221 patients who underwent TACE in the Department of Intervention, Union Hospital, Tongji Medical College, Huazhong University of Science and Technology from January 2019 to December 2020 were collected. Inclusion criteria (1) Clinical or pathological diagnosis of primary hepatocellular carcinoma; (2) TACE treatment ≤2 times; (3) Child-Pugh classification of liver function [13] A or B, performance status score (ECOG) 0–1; (3) Aged 18–70 years old; (4) No use of molecular targeted drugs or immunotherapy; (5) No central nervous system disease, intestinal obstruction and other primary diseases that can lead to nausea and vomiting. Exclusion criteria: (1) Child-Pugh classification of liver function C, performance status score (ECOG) ≥ 2; (2) severe coagulopathy and can not be corrected; (3) cachexia or extensive distant metastasis of the tumor; (4) complete portal vein occlusion and collateral vessels; (5) renal insufficiency.

### Method

Routine preparation, disinfection, draping. After local anesthesia with 2% lidocaine, the right femoral artery was punctured using the Seldinger technique and a 5F vascular sheath was placed. The feeding artery of the tumor was identified by catheterization with a 5F Yashino catheter to the celiac trunk and superior mesenteric artery for angiography. Then 2.7F microcatheter was superselectively cannulated into the tumor feeding artery, appropriate amount of Lipiodol + chemotherapeutic emulsion was slowly injected for embolization, and 300–500 um gelatin sponge particles were supplemented for embolization. The embolization endpoint was forward blood flow stasis in the tumor feeding artery. Chemotherapeutic drugs used during surgery are divided into two types: (1) lobaplatin 50 mg; (2) epirubicin 30 mg. The amount of iodized oil used was 5–20 ml.Evaluation criteria for nausea and vomiting: Common Terminology Criteria for Adverse Events (CTCAE 5.0).

Materials and drugs used for TACE: 5F vascular sheath (TERUMO5F-10CM, Terumo, Japan), 0.035 in. (RFGA35153M, Terumo, Japan), 5F Yashino catheter (Terumo, Japan), 2.7F microcatheter (Terumo, Japan). Lobaplatin (GYZZ H20050308, Hainan Chang’an International Pharmaceutical Co., Ltd.), epirubicin (GYZZ H19990280, Zhejiang Hisun Pharmaceutical Co., Ltd.).

SPSS software (Version 24.0, IBM, Armonk, NewYork) was used to statistically analyze the data. Enumeration data are expressed by number of cases (percentage). Measurement data were expressed as mean ± standard deviation. The models are multivariate, Binary logistic regression analysis was used, method used Enter, the indicator (first) was used for comparison, and *P* < 0.05 was considered statistically significant.

## Results

### Basic information

There were 136 (61.5%) male patients and 85 (38.5%) female patients. The age ranged from 24 to 69 years, with an average of 47.9 ± 12.0 years. Seventeen patients (7.7%) were aged ≤30 years, 133 (60.2%) were aged 30–55 years, and 71 (32.1%) were aged ≥55 years.

**Fig. 1:**
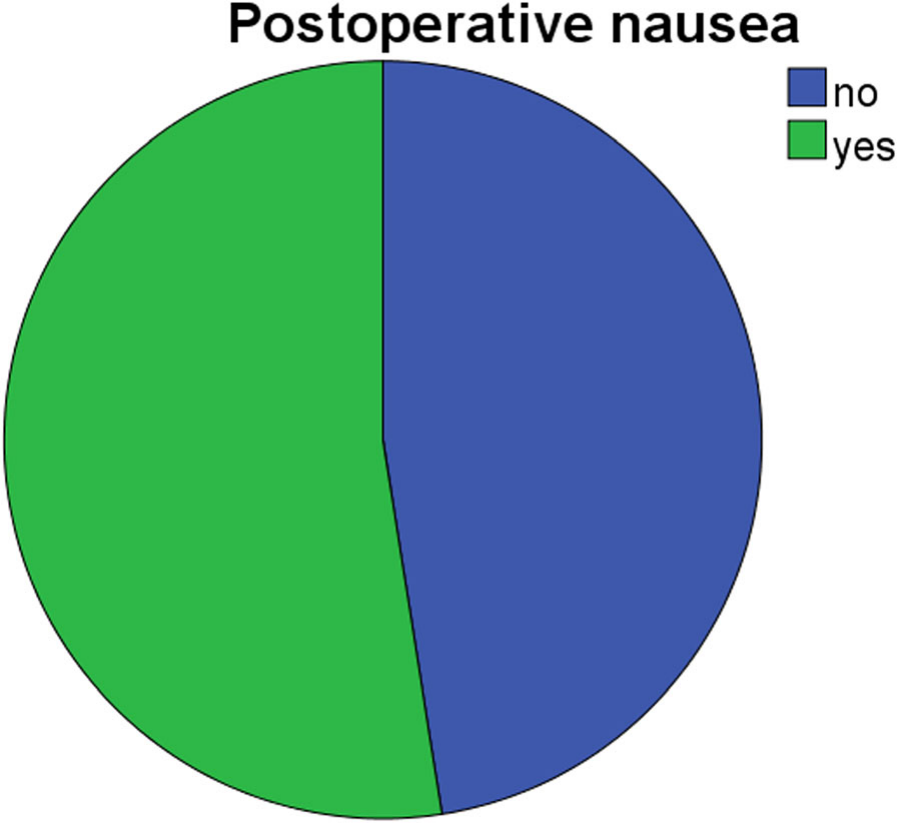
Postoperative nausea occurred in 116 patients (52.5%)

**Fig. 2:**
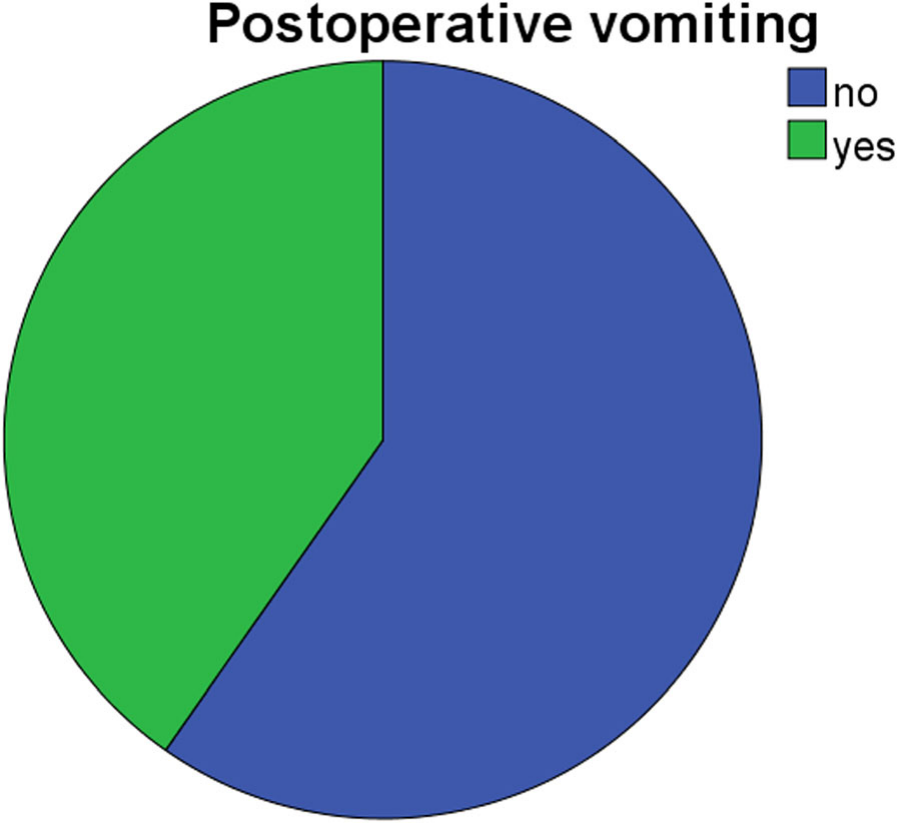
Postoperative vomiting occurred in 89 patients (40.3%), and patients with postoperative vomiting were accompanied by nausea

Preoperative Child-Pugh classification of liver function: 157 patients (71%) in class A and 64 patients (29%) in class B. ALT: 11–96 U/L, with an average of 44.2 ± 19.0 U/L. AST: 8–86 U/L, with an average of 42.0 ± 17.4 U/L. ALP: 40–198 U/L, with an average of 92.0 ± 26.8 U/L (Table 1). The causes of cirrhosis were hepatitis B in 196 patients (88.7%) and hepatitis C in 25 patients (11.3%). BCLC stage (12): 29 patients (13.1%) in stage A, 123 patients (55.7%) in stage B, and 69 patients (31.2%) in stage C. Preoperative analgesic drugs were used in 64 patients (29%) and no analgesic drugs were used in 157 patients (71%). Preoperative limosis was performed in 111 patients (50.2%) and non-limosis in 110 patients (49.8%). Forty patients (18.1%) had a previous history of vomiting and 181 patients (81.9%) had no history of vomiting. Forty-four patients (19.9%) had a history of kinetosis and 177 patients (80.1%) had no history of kinetosis. Forty-two patients (19%) had a smoking history and 179 patients (81%) had no smoking history. Forty-nine patients (22.2%) had a history of alcohol consumption and 172 patients (77.8%) had no history of alcohol consumption. Chemotherapeutic drugs were used during surgery: lobaplatin in 109 patients (49.3%) and epirubicin in 112 patients (50.7%). The amount of lipiodol used was: < 5 ml in 81 patients (36.7%), 5–10 ml in 85 patients (38.5%), and > 10 ml in 55 patients (24.9%). Postoperative patients had pain in 109 patients (49.3%) (Table 2). Postoperative nausea occurred in 116 patients (52.5%). Postoperative vomiting occurred in 89 patients (40.3%), and patients with postoperative vomiting were accompanied by nausea (Table 3) (Figs. 1 and 2).

**Table 1:** Descriptive statistics

	Minimum	Maximum	Mean	Std. Deviation
**Age**	24	69	47.92	11.998
**ALT(U/L)**	11	96	44.22	19.015
**AST(U/L)**	8	86	42.06	17.366
**ALP(U/L)**	40	198	92.00	26.774

**Table 2:** General information of patients

	Frequency	Percent
**Gender**		
Female	112	50.7
Male	109	49.3
**Age group**		
≤30	17	7.7
30–55	133	60.2
≥55	71	32.1
**Child-Pugh Classification**		
A	157	71.0
B	64	29.0
**Etiology of liver cirrhosis**		
Hepatitis B	196	88.7
Hepatitis C	25	11.3
**BCLC stage**		
A	29	13.1
B	123	55.7
C	69	31.2
**History of vomiting**		
No	181	81.9
Yes	40	18.1
**Kinetosis**		
No	177	80.1
Yes	44	19.9
**Smoking history**		
No	179	81.0
Yes	42	19.0
**Alcohol history**		
No	172	77.8
Yes	49	22.2
**Use of analgesics**		
No	157	71.0
Yes	64	29.0
**Chemotherapeutic drugs**		
Lobaplatin	109	49.3
Epirubicin	112	50.7
**Dosage of Lipiodol**		
< 5 ml	81	36.7
5-10 ml	85	38.5
> 10 ml	55	24.9
**Limosis before operation**		
No	110	49.8
Yes	111	50.2
**Postoperative pain**		
No	112	50.7
Yes	109	49.3

**Table 3:** Incidence of nausea and vomiting after TACE

	Frequency	Percent
**Postoperative nausea**		
No	105	47.5
Yes	116	52.5
**Postoperative vomiting**		
No	132	59.7
Yes	89	40.3

### Incidence of nausea after TACE

Nausea occurred in 116 patients (52.5%) after TACE. Binary logistic regression analysis (Table 4) was used, with the method using enter and the contrast using indicator (first).

**Table 4:**
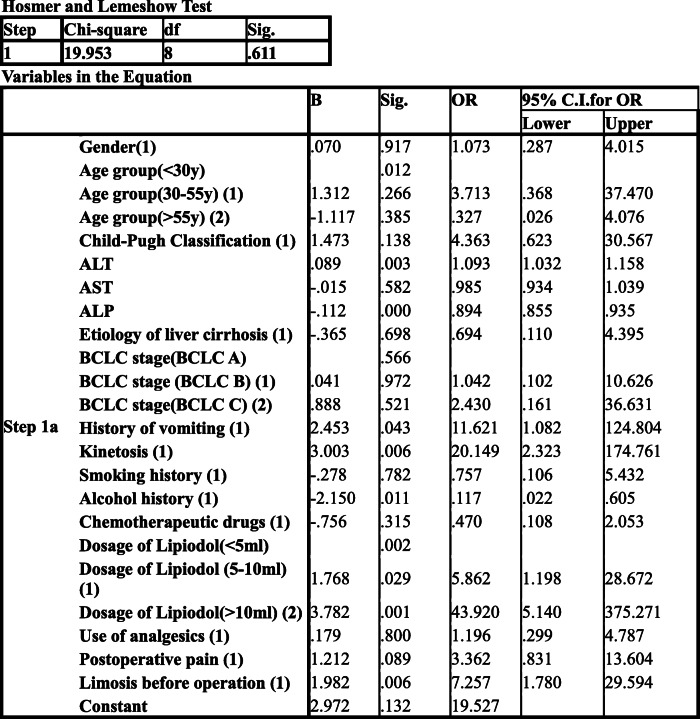
Postoperative nausea after TACE, Binary logistic regression analysis

Sig(*P* value): ALT0.003; ALP0.000; history of vomiting 0.043; kinetosis 0.006; history of alcohol consumption 0.011; preoperative limosis 0.006; dosage of lipiodol (5–10 mL) 0.029, dosage of lipiodol (> 10 mL) 0.001. The above are risk factors for post-TACE nausea. (*P* < 0.05) OR values, Exp (B): ALT1.093; ALP0.894; history of vomiting 11.621; kinetosis 20.149; history of alcohol consumption 0.117; preoperative limosis 7.257; dosage of lipiodol (5–10 mL) 5.862, dosage of lipiodol (> 10 mL) 43.920.

### Incidence of vomiting after TACE

Vomiting occurred in 89 patients (40.3%) after TACE, all of whom had nausea. Binary logistic regression analysis (Table 5) was used, with the method using enter and the contrast using the indicator (first).

**Table 5:**
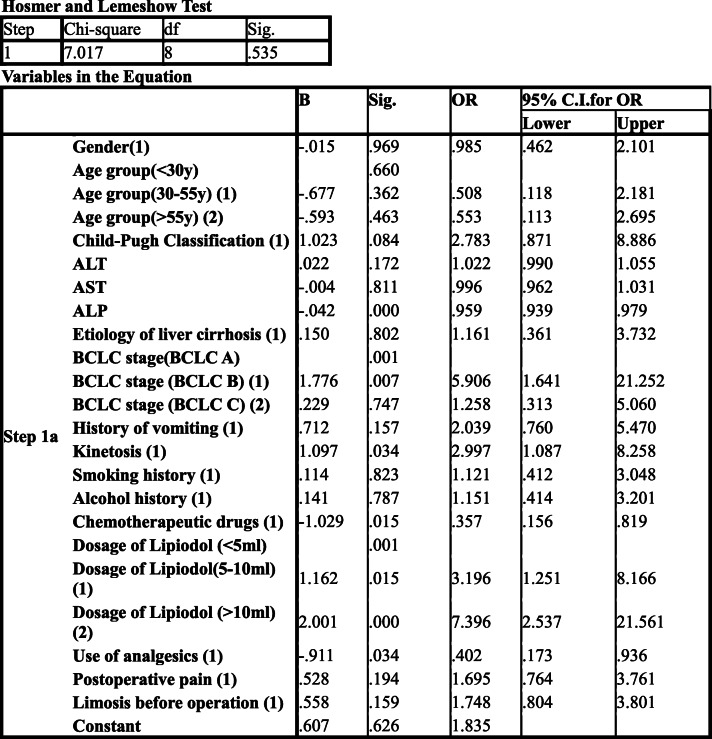
Postoperative vomiting after TACE, Binary logistic regression analysis

Sig(*P* value): ALP0.000; BCLC stage (B) 0.007; kinetosis 0.034; chemotherapeutic drugs 0.015; dosage of lipiodol (5–10 ml) 0.015, dosage of lipiodol (> 10 ml) 0.000; patients used analgesics before TACE 0.034. The above are risk factors for nausea and vomiting after TACE. (*P* < 0.05) OR values, Exp (B): ALP0.959; BCLC stage (B) 5.906; kinetosis s 2.997; chemotherapeutic drugs 0.357; dosage of lipiodol (5–10 ml) 3.196, dosage of lipiodol (> 10 ml) 7.396; patients used analgesics before TACE 0.402.

## Discussion

Nausea is a feeling of visceral discomfort that eventually reaches the climax of the response with vomiting. Vomiting is a protective reflex that refers to the process by which gastric contents are excreted from the body through the mouth. A variety of receptors have been found to trigger nausea and vomiting [14, 15]. (1) Visceral receptors: Visceral receptors are divided into mechanical and chemical receptors. Mechanical receptors are located in the gastrointestinal wall and are sensitive to traction stimulation of the gastrointestinal wall. Chemoreceptors are mainly located in the gastrointestinal mucosa and can sense changes in the internal environment of the digestive tract, irritation by drugs and poisons [16]. (2) Receptors of the area postrema: The area postrema is located on the dorsal surface of the medulla oblongata and at the level of the base latch of the fourth ventricle. This structure is abundant and lacks the blood-brain barrier and the cerebrospinal fluid-brain barrier. Chemicals, certain peptides in the body, etc., can act on receptors in the area postrema to cause vomiting. (3) Vestibular Receptors: Vestibular receptors are abnormally stimulated (motor stimulation, position change) and can cause nausea and vomiting, but studies have shown that there is insufficient evidence that vestibular receptors play a direct role in vomiting caused by drug stimulation. (4) Descending signals in higher centers: Studies have found that certain psychiatric factors, hypotension, pain, increased intracranial pressure, and craniocerebral injury act descending on vomiting centers through different regions of the cerebral cortex, causing nausea and vomiting [17]. The mechanism of nausea and vomiting is not fully understood, but a variety of transmitters and receptors are known to be involved in this process, such as acetylcholine, epinephrine, norepinephrine, dopamine, histamine, serotonin, substance P, Y-aminobutyric acid, and opioid receptors [18, 19].

### Postoperative nausea and vomiting (PONV)

PONV is a common complication after surgical procedures, with an incidence of approximately 30% [20]. Without prevention, the incidence of PONV in the population is as high as 80% [21]. Common causes of PONV are: (1) anesthetic factors. The use of inhaled anesthetics and opioids, is an important factor causing PONV. (2) Surgical factors. Surgical trauma and inflammatory reactions, which can cause the release of serotonin, substance P, and other transmitters, result in nausea and vomiting. Studies have shown that the longer the operation time, the higher the risk of PONV. The type of surgery is also one of the causes of PONV, such as hepatobiliary surgery, gynecological surgery, and otolaryngological surgery are more likely to develop PONV than other types of surgery [22]. (3) Patient factors. Young women, no smoking history, history of PONV, and history of kinetosis were risk factors confirmed by the study. Other possible risk factors are BMI, American Society of Anesthesiologists (ASA) classification, history of migraine, and menstrual cycle [23].

### Chemotherapy-induced nausea and vomiting (CINV)

Chemotherapy plays an important role in the treatment of malignant tumors, and CINV is one of the common adverse reactions of chemotherapy. Chemotherapeutic drugs can act directly on the intestinal mucosa through the intestinal lumen, or activate the chemoreceptor trigger zone through the blood circulation, which promotes the release of a variety of neurotransmitters and acts on the vomiting center, resulting in nausea and vomiting [24, 25]. The National Comprehensive Cancer Network classifies emetogenic antineoplastic agents into four levels [26], and different antineoplastic agents, have different emetogenic risks.

### Mechanism of nausea and vomiting after TACE

Similar to surgery, TACE is an invasive treatment that may cause pain and inflammatory reaction of organs, and patients may use analgesics. The differences between TACE and surgical operation include: (1) TACE is usually a local anesthesia operation, which is generally a puncture site injection of local anesthetics and does not require the use of inhaled anesthetics; (2) TACE usually uses chemotherapeutic drugs; chemotherapeutic drugs are generally not used through vascular route during surgery; (3) TACE embolizes tumor feeding artery, which will lead to tumor ischemia, hypoxia, necrosis and aseptic inflammation; while surgical operation includes organ trauma and traction. Similar to chemotherapy, chemotherapeutic agents will be used during TACE. However, the dose of chemotherapeutic drugs used during surgery was significantly lower in TACE than in chemotherapy. Moreover, compared with chemotherapy, TACE performed embolization of tumor vessels, resulting in ischemia of the tissue. Because of the differences between TACE and surgery and chemotherapy, the causes of nausea and vomiting after TACE are not exactly the same as those of PONV and CINV. We believe that the causes of nausea and vomiting after TACE include: (1) trauma caused by operation, aseptic inflammation caused by ischemia and hypoxia, inducing the release of a variety of transmitters; (2) intraoperative use of chemotherapeutic drugs, through the blood circulation, stimulating the chemoreceptors of the gastrointestinal tract; especially during TACE, chemotherapeutic drugs are often injected through the celiac trunk or superior mesenteric artery branches, while the celiac trunk or superior mesenteric artery has vascular branches directly supplying the gastrointestinal tract; epirubicin and platinum used during TACE are chemotherapeutic drugs with moderate to high emetic risk; (3) the organs involved in TACE are the liver and gallbladder, and hepatic artery embolization will lead to hepatobiliary ischemia; similar to the causes of PONV, hepatobiliary surgery is more likely to have postoperative nausea and vomiting than other surgeries; (4) stress and pain during TACE will lead to the release of dopamine, epinephrine and other transmitters, leading to nausea and vomiting; (5) patient factors, such as young women, no history of smoking, history of PONV, and history of kinetosis [27].

### Risk factors of nausea and vomiting after TACE

Binary logistic regression analysis showed that ALP, BCLC stage, kinetosis, chemotherapeutic drugs, dosage of lipiodol and preoperative use of analgesics were risk factors affecting nausea and vomiting after TACE. Similar to PONV and CINV, the history of kinetosis was a risk factor for nausea and vomiting after TACE [28], and the OR for nausea and vomiting after TACE was 2.997 in patients with a history of kinetosis relative to those without a history. Compared with BCLC A stage, BCLC B stage has higher risk of postoperative nausea and vomiting, and may be multinodular liver cancer or massive liver cancer in BCLC B stage, with more dosage of embolic agent and wider embolization range. Lipiodol dose is a risk factor for nausea and vomiting after TACE. It was considered that the more lipiodol is used, the wider the embolization range, the more severe the tissue ischemia and hypoxia, aseptic inflammation and more transmitters are released. Kabuki M [29] etal shows that the multivariate logistic regression model with a predictive success of 92. 4% for vomiting identified significant associations between female gender (odds ratio: 3.73, *p* < 0.001), the number of tumors (1.29, *p* < 0.01), and administration of pentazocine (11.70, *p* < 0.05) with the risk of vomiting. The results of this study showed that chemotherapeutic drugs were the risk factors of postoperative nausea and vomiting after TACE. The incidence of postoperative nausea and vomiting was higher in patients who used lobaplatin during the operation. Several studies showed that the use of lobaplatin emulsion for embolization resulted in a higher tumor necrosis rate. Possibly, due to this reason, the more necrotic substances were released after TACE, the more severe the inflammatory response, and the risk of postoperative nausea and vomiting. The results showed that ALP was a risk factor for nausea and vomiting after TACE. The risk of postoperative nausea and vomiting in patients with low ALP level was consistent with the results of Wang [27], Wang etal.found out patients who developed vomiting, compared to those who did not, also had lower levels (< 100 IU/L) of serum ALP (112.52 ± 62.63 vs. 160.10 ± 127.80, respectively, *p* = 0.010), and serum alanine transferase (ALT) (35.61 ± 22.87 vs. 44.97 ± 29.62, respectively, *p* = 0.045). wang etal. Observed that lower levels of ALP (< 100 IU/L) occurred more frequently in patients with lower levels of ALT, AST and LDH (*p* = 0.002, *p* = 0.000 and *p* = 0.001, respectively). But why patients with better liver function are more likely to experience nausea and vomiting after TACE, the reason for this remains unclear. Preoperative use of analgesic drugs was the risk factor of nausea and vomiting after TACE (OR = 0.402), which was inconsistent with PONV. The reason was analyzed that in this group of patients with hepatocellular carcinoma, oral drugs were used for analgesia, and non-steroidal anti-inflammatory drugs were mainly used. Non-steroidal anti-inflammatory drugs could reduce the aseptic inflammation caused by postoperative tumor necrosis. At the same time, good analgesia could reduce the stress response of patients, so the risk of postoperative nausea and vomiting was lower.

## Conclusions

There were multiple causes for nausea and vomiting after TACE, including operative trauma, aseptic inflammation caused by ischemia and hypoxia, intraoperative use of chemotherapeutic drugs, ischemia of liver and bile duct, stress and pain during TACE, and patient factors (such as young women, no smoking history, history of PONV, and history of kinetosis). The above are all possible causes of nausea and vomiting after TACE, and among these factors, ALP, BCLC stage, chemotherapeutic drugs, dosage of lipiodol, and preoperative patient use of analgesics were risk factors affecting nausea and vomiting after TACE. Careful evaluation of patients’ factors before TACE and early preventive intervention can reduce patients’ psychological and physical burden, reduce perioperative risks, improve patients’ treatment compliance and reduce patients’ hospitalization costs.

The shortcomings of this study were that the data were from a single center and the sample size was limited. A multi-center and large-sample study was feasible in the later stage. The risk assessment table of nausea and vomiting before TACE could be developed according to the risk factors in the later stage to provide more help for clinical work.

## Data Availability

The datasets used and analysed during the current study are available from the corresponding author on reasonable request.
